# Anti-Inflammatory Potential of Monogalactosyl Diacylglycerols and a Monoacylglycerol from the Edible Brown Seaweed *Fucus spiralis* Linnaeus

**DOI:** 10.3390/md12031406

**Published:** 2014-03-11

**Authors:** Graciliana Lopes, Georgios Daletos, Peter Proksch, Paula B. Andrade, Patrícia Valentão

**Affiliations:** 1 REQUIMTE/Laboratory of Pharmacognosy, Department of Chemistry, Faculty of Pharmacy, University of Porto, Rua de Jorge Viterbo Ferreira 228, Porto 4050-313, Portugal; E-Mails: gracilianalps@gmail.com (G.L.); pandrade@ff.up.pt (P.B.A.); 2 Institute of Pharmaceutical Biology and Biotechnology, Heinrich-Heine University, Universitätsstraße 1, Düsseldorf 40225, Germany; E-Mail: georgios.daletos@uni-duesseldorf.de

**Keywords:** *Fucus spiralis*, monoacylglycerol, monogalactosyl diacylglycerols, anti-inflammatory, LC-MS, NMR

## Abstract

A monoacylglycerol (**1**) and a 1:1 mixture of two monogalactosyl diacylglycerols (MGDGs) (**2** and **3**) were isolated from the brown seaweed *Fucus spiralis* Linnaeus. The structures were elucidated by spectroscopic means (NMR and MS) and by comparison with the literature. Compound **1** was composed of a glycerol moiety linked to oleic acid (C18:1 Ω9). Compounds **2** and **3** contained a glycerol moiety linked to a galactose unit and eicosapentaenoic acid (C20:5 Ω3) combined with octadecatetraenoic acid (C18:4 Ω3) or linolenic acid (C18:3 Ω3), respectively. The isolated compounds were tested for their cytotoxic and anti-inflammatory activity in RAW 264.7 macrophage cells. All of them inhibited NO production at non-cytotoxic concentrations. The fraction consisting of compounds **2** and **3**, in a ratio of 1:1, was slightly more effective than compound **1** (IC_50_ of 60.06 and 65.70 µg/mL, respectively). To our knowledge, this is the first report of these compounds from *F. spiralis* and on their anti-inflammatory capacity.

## 1. Introduction

Glyceroglycolipids are the most widespread lipids in nature. These amphiphatic compounds constitute a structurally heterogeneous group with functional roles in several biological systems [1]. They are synthesized by all eukaryotic and prokaryotic organisms and constitute important membrane lipids with a key role in energy storage, in membrane formation and fluidity and in chemical interactions with the environment [2]. Within this group of metabolites one can find mono and digalactosyl diacylglycerols (MGDGs/DGDGs), which are the major constituents of photosynthetic membranes [1]. These compounds consist of a glycerol unit linked to one (MGDGs) or two (DGDGs) molecules of galactose and one or two fatty acids, mainly unsaturated at Ω3 [3]. MGDGs and DGDGs constitute the most widespread non-phosphorous polar lipids in nature, accounting for about 80% of membrane lipids in green plant tissues and more than half of all lipids in algae [2,3]. Beyond their structural and chemical functions, these compounds present several promising biological properties, among which the anti-tumor [4,5], anti-viral [6], algicidal [7] and anti-inflammatory [8,9,10,11] activities can be highlighted.

Inflammation is a pathological condition in which highly reactive species are produced. Nitric oxide (NO) is a small diffusible molecule responsible for vasodilatation, neurotransmission and inflammation. This molecule is produced by the organism at a basal concentration. Nevertheless, under stimulation by pathogens, NO is generated in higher amounts by the inducible nitric oxide synthase (iNOS) in activated macrophages. The overproduction of NO is involved in the pathogenesis of septic shock, tissue damage, multiple organ dysfunctions and carcinogenesis processes [12]. The anti-inflammatory activity and the capacity to sequester free radicals by extracts and isolated compounds from marine organisms have attracted researchers’ attention in the past few years. As the majority of marine organisms, seaweeds constitute an endless source of bioactive secondary metabolites with promising effects for human health [13].

The brown seaweed *Fucus spiralis* Linnaeus is a widely distributed species over the Portuguese west coast. This edible seaweed is rich in secondary metabolites, such as phlorotannins, sterols and fatty acids, which are associated with several biological activities, namely antioxidant, anti-allergic, antimicrobial, anti-ageing and anti-diabetic activities [14,15,16,17,18]. Among the secondary metabolites produced by this species, fatty acids constitute a very special group. *F. spiralis* has a diverse lipid composition, dominated by mono and polyunsaturated long chain fatty acids, oleic acid being the major one [18]. This fatty acid, together with arachidonic and eicosapentaenoic acids, has been reported to inhibit acetylcholinesterase in a dose-dependent manner [19,20]. Although there are already some studies on the lipid composition of this species, as far as we know the glyceroglycolipids have not been explored yet.

The beginning of compounds isolation has revolutionized world’s medicine and promoted the exponential growth of the pharmaceutical industry. For the first time, specific pharmacological activities could be attributed to specific compounds and groups of compounds, thanks to spectroscopic and chromatographic methodologies. In this work, we describe the isolation and identification of glyceroglycolipids from *F. spiralis* and their NO inhibitory activity in lipopolysaccharide (LPS) stimulated RAW 264.7 macrophages. As far as we are aware, glyceroglycolipids are being described in *F. spiralis* for the first time. Additionally, this is the first report concerning the anti-inflammatory activity of the characterized compounds.

## 2. Results and Discussion

### 2.1. Isolation of Monoacylglycerol and Monogalactosyl Diacylglycerols

**Figure 1:**
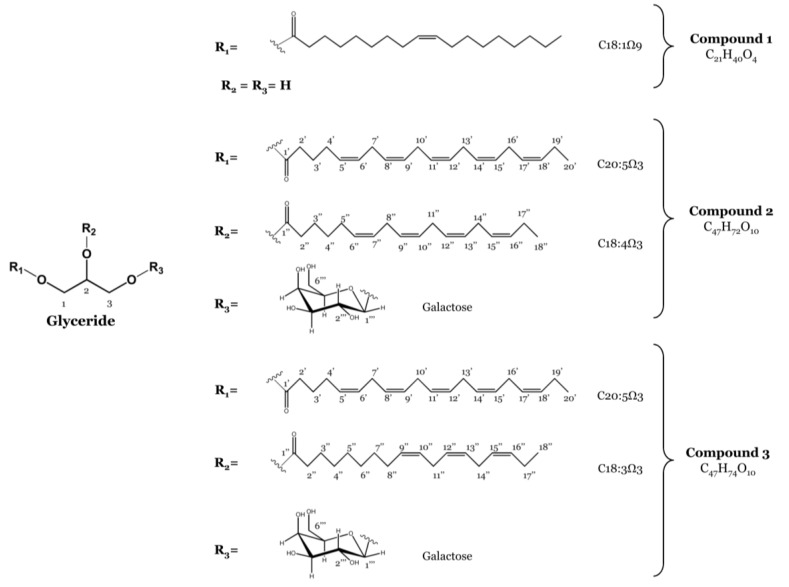
Structures of the monoacylglycerol (Compound **1**) and the monogalactosyldiacylglycerols (MGDGs) (Compounds **2** and **3**) isolated from *F. spiralis*.

The concentrated methanolic extract of *F. spiralis* was subjected to solvent-solvent partitioning to give n-hexane, EtOAc and *n*-BuOH fractions. The EtOAc fraction was partitioned as outlined in the experimental section to yield one monoacylglycerol featuring oleic acid (C18:1 Ω9) (**1**) and a 1:1 mixture of two MGDGs containing eicosapentaenoic acid (C20:5 Ω3) combined with octadecatetraenoic acid (C18:4 Ω3) (**2**) or linolenic acid (C18:3 Ω3) (**3**), respectively (Figure 1). Their structures and the regiochemical attachments of the acyl chains to the glycerol moiety were unambiguously elucidated on the basis of extensive NMR spectroscopic analysis and mass spectrometry and by comparison with data reported in the literature [7,21]. Previous reports include the isolation of **2** and **3** from the brown seaweed *Sargassum thunbergii* (Mertens ex Roth) Kuntze [21], the marine dinoflagellate *Heterosigma akashiwo* (Y. Hada) Y. Hada ex Y. Hara & M. Chihara [22] and from the freshwater dinoflagellate *Glenodinium sanguineum* (H. J. Carter) Diesing [23] and their detection in glaucocystophytes [24].

### 2.2. Anti-Inflammatory Activity

#### 2.2.1. Cell Viability

**Figure 2:**
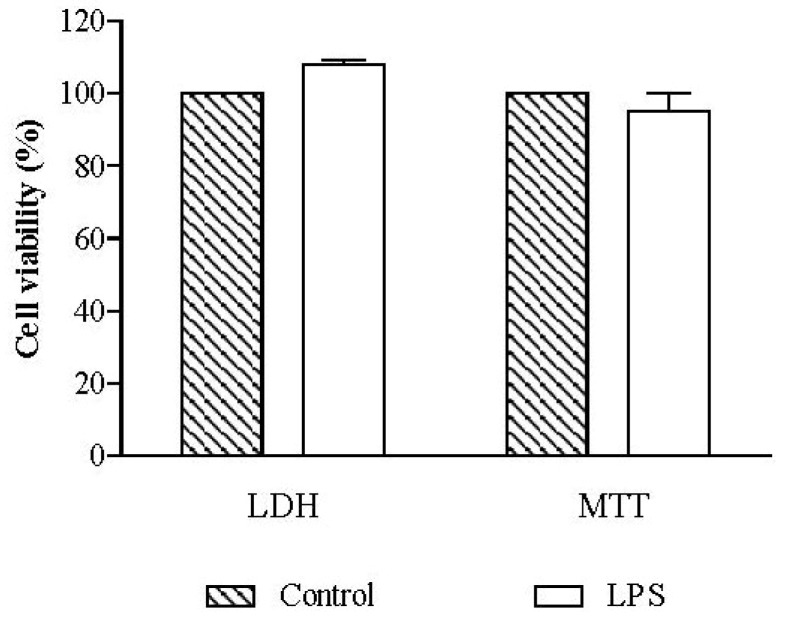
Influence of lipopolysaccharide (LPS) in cell viability. RAW 264.7 macrophages were exposed to 1 µg/mL of LPS during 18 h and cell viability was assessed by lactate dehydrogenase (LDH) and thiazolyl blue tetrazolium bromide (MTT) assays. Results are expressed in percentage of control (mean ± SEM of four independent assays performed in duplicate).

The viability of RAW 264.7 macrophages was evaluated using non-treated cells, in order to assess the effect of 18 h of exposure to 1 µg/mL LPS. Cell viability, evaluated by the lactate dehydrogenase (LDH) and thiazolyl blue tetrazolium bromide (MTT) assays, was not significantly different from that of non-exposed control cells (Figure 2).

**Figure 3:**
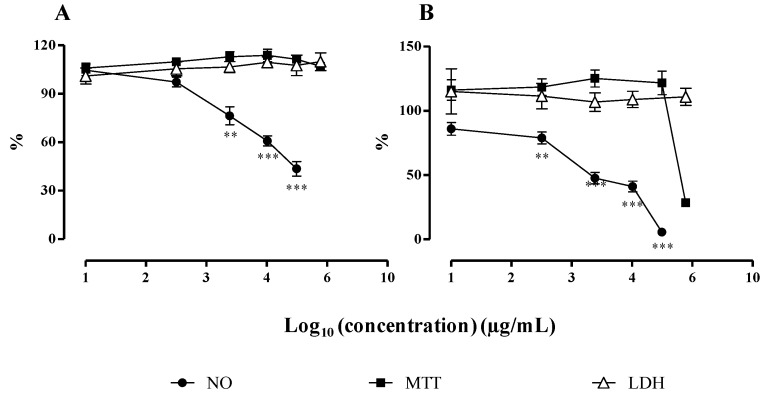
Influence of the monoacylglycerol (**A**) and the MGDGs (**B**) on cell viability and on NO released by macrophages. After pre-exposure with the test compounds and stimulation with LPS, the viability of RAW 264.7 cells was assessed by the LDH and MTT assays, and the NO production was quantified. Results are expressed as percentage of control (mean ± SEM of four independent assays performed in duplicate). ** *p* < 0.01, *** *p* < 0.001.

The cytotoxicity of the monoacylglycerol (**1**) and of the fraction containing MGDGs (**2** and **3**), in a ratio of 1:1, isolated from *F. spiralis* was also evaluated by the MTT and LDH assays, prior to testing them for their anti-inflammatory activity (Figure 3).

Concerning the monoacylglycerol, exposure to LPS had no effect on cell viability at the tested concentrations (6.25–100 µg/mL). There was no statistical difference relative to control for both MTT and LDH assays (Figure 3), and the microscopic evaluation revealed normal size and shape of the cells. The same pattern was observed in control cells, treated with the monoacylglycerol but without LPS.

On the other hand, cells treated with the highest concentration of the MGDGs fraction (500 µg/mL) suffered a decrease in cell viability to 33.06% ± 3.68%, relative to control, in MTT assay (*p* < 0.001) (Figure 3). This assay, which provides information about the mitochondrial function of the cells by evaluating succinate dehydrogenase activity, proves that there was a drastic loss of viability [25]. Curiously, this effect was not confirmed with the LDH assay, which presented values around 100% relative to control for all the tested concentrations (Figure 3). The lack of LDH in the extracellular medium indicates that there was no cell membrane damage.

In order to confirm these results, cells were observed using light microscopy, immediately before starting with the treatment (this is, before pre-exposing the cells for 1 h to the tested compounds) and after the established incubation period (1 h exposure to the tested compounds followed by 18 h incubation with both test compounds and LPS). This allowed us to observe that before exposure to test compounds and 1 h after exposure, macrophages presented their normal size and shape. After 18 h of co-treatment with LPS, cells presented severe modifications in size, shape and density for the highest concentration of MGDGs tested (500 µg/mL).

Face to this observations, we may consider that, at the cytotoxic concentration of 500 µg/mL, the tested MGDGs promoted the death of the majority of cells. This observation is not supported by the results of the LDH assay (which indicate cell viability *per se*), but was previously observed and confirmed by other authors using the same cell line, treated in a similar manner [26]: while assessing the anti-inflammatory potential of plumbagin on LPS-stimulated Raw 264.7 macrophage cells Pinho and colleagues verified that the LDH could be released from macrophages in an early stage of the treatment and then degraded during the incubation period, reaching values close to control [26]. Moreover, the culture medium Dulbecco’s Modified Eagle Medium (DMEM) retained its color very close to the initial one, which did not happened in the other wells. DMEM has a pH indicator that changes from red to yellow by acidification, the pH decrease being characteristic of cell metabolism. The fact that no change of the color of the culture medium was observed is another indicative of cell death or metabolic disability.

#### 2.2.2. NO Released by RAW 264.7 Macrophages

NO is a diffusible free radical with many functions in diverse biological systems. It is synthesized from arginine by nitric oxide synthase (NOS) and acts as an important inflammatory mediator. iNOS is an inducible form of NOS, which is induced under pathological conditions, being responsible for the overproduction of NO [27].

**Figure 4:**
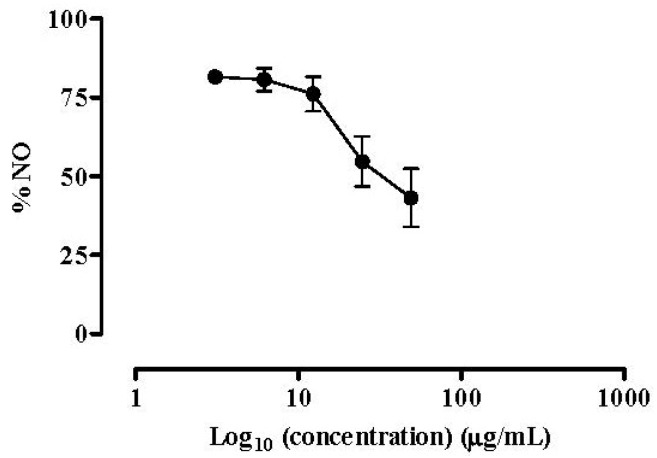
Dexamethasone effect on NO release. Quantification of NO produced by RAW 264.7 cells exposed to dexamethasone and stimulated with 1 µg/mL of LPS for 18 h. Results are expressed in percentage of control with LPS (mean ± SEM of four independent assays performed in duplicate).

The isolated compounds were screened for their ability to affect NO released by LPS stimulated RAW 264.7 macrophages. All of them showed a dose-dependent NO inhibitory activity. The monoacylglycerol, composed of a glycerol moiety linked to oleic acid (C18:1 Ω9) (**1**), demonstrated lower capacity to inhibit NO production by macrophages than the 1:1 mixture of the MGDGs (**2** and **3**) (IC_50_ = 65.70 µg/mL *vs*. 60.06 µg/mL, respectively). The anti-inflammatory reference drug dexamethasone was used as positive control, as inhibitor of NO production by macrophages (Figure 4). Macrophage cells were treated with dexamethasone along with the isolated compounds. Like the isolated compounds, the reference anti-inflammatory drug was able to inhibit NO production in a dose-dependent manner (IC_50_ = 34.60 µg/mL) (Figure 4).

According to previous studies [11,28], it is known that galactolipids are able to down-regulate the iNOS protein levels in LPS-stimulated RAW 264.7 cells, suggesting that the decrease in NO production observed for these compounds is related to the down regulation of iNOS expression [11,28]. The authors evaluated the capacity of two MGDGs composed of hexadecatetraenoic acid (C16:4 Ω3) combined with octadecatetraenoic (C18:4 Ω3) and linolenic (C18:3 Ω3) acids to reduce NO production in RAW 264.7 cells and concluded that the compound with more double bonds presented a lower IC_50_, proving that the levels of insaturation are related to the anti-inflammatory activity. The same research group reported the NO inhibitory capacity of two MGDGs composed by one glycerol unit linked to a fatty acid and one molecule of galactose. With this study they concluded that the position of the double bonds was more crucial for the bioactivity than the number of double bonds, since compounds with Ω6 fatty acids were more active than Ω3 fatty acids (even having more double bonds) [11,28].

Those findings are in accordance with our results, as both the monoacylglycerol and the MGDGs showed the capacity to reduce NO release in a dose-dependent manner. According to the previous literature [11] the difference between the IC_50_ values of MGDGs fraction and the monoacylglycerol can be justified by the position of the double bond in the monoacylglycerol at Ω9.

Although the IC_50_ values of our compounds are higher than that of the reference drug, the potential of the monoacylglycerol and of the MGDGs as anti-inflammatory agents cannot be set aside. Glucocorticoids are commonly prescribed for the treatment of several situations of inflammatory disorders; nevertheless, there are plenty of undesirable side effects associated with both acute and long term treatments [29]. For this reason, we believe that glycerolipids and MGDGs may constitute an alternative to common anti-inflammatory drugs, as they present a good therapeutic activity without known side effects.

The anti-inflammatory activity of this kind of compounds has been more extensively studied on cells from articular cartilage [9,30]. MGDGs have shown a potent anti-inflammatory activity in *in vitro* cultured articular chondrocytes and also the ability to prevent cell proliferation, without affecting cell viability, during osteoarthritic cartilage degeneration [9,30]. Maeda and colleagues went further and investigated the action of MGDGs on colon tumor in mice after an oral administration, demonstrating that these compounds were safe and had a potent antitumor effect [5]. These compounds also proved to be able to inhibit croton-oil-induced ear edema in the mouse, with a better IC_50_ than the reference drug [8].

## 3. Experimental Section

### 3.1. Standards and Reagents

β-Nicotinamide adenine dinucleotide reduced form (NADH), dexamethasone, sodium pyruvate, 3-(4,5-dimethyl-2-thiazolyl)-2,5-diphenyl2H-tetrazolium bromide (MTT), sulphanilamide, dimethyl sulfoxide (DMSO), *p*-anisaldehyde, dichloromethane and ethyl acetate and lipopolysaccharide (LPS) from *Salmonella enterica* were from Sigma-Aldrich (Steinheim, Germany). Sulphuric acid (H_2_SO_4_), *ortho*-phosphoric acid (H_3_PO_4_) and glacial acetic acid were from Panreac (Barcelona, Spain). HPLC-grade methanol, acetonitrile, *N*-(1-naphthyl) ethylenediamine and silica gel 60 M F254, were obtained from Merck (Darmstadt, Germany). DMEM, Dulbecco’s phosphate buffered saline (DPBS), heat inactivated foetal bovine serum (FBS) and Pen Strep solution (Penicillin 5000 units/mL and Streptomycin 5000 mg/mL) were purchased from Gibco (Invitrogen, Paisley, UK). The murine macrophage-like cell line RAW 264.7 was from the American Type Culture Collection (ATCC, LGC Standards S.L.U., Barcelona, Spain) and was kindly provided by Maria S. J. Nascimento (Laboratório de Microbiologia, Departamento de Ciências Biológicas, Faculdade de Farmácia, Universidade do Porto). Water was deionized using a Milli-Q water purification system (Millipore, Bedford, MA, USA).

### 3.2. Compounds Characterization

#### 3.2.1. General Experimental Methods

^1^H, ^13^C and 2D NMR spectra were recorded at 25 °C on a Bruker DRX 500 NMR spectrometer. Mass spectra (ESI) were recorded with a HP1100 Agilent Finnigan LCQ Deca mass spectrometer. HPLC analysis was performed using a Dionex UltiMate 3000 System coupled to a photodiode array detector (UVD340S), with routine detection wavelengths at 235, 254, 280 and 340 nm. The chromatographic analysis was carried out on a Knauer VertexPlus column (125 × 4 mm, Eurospher 100-10, C18). The mobile phase consisted of two solvents: 0.1% phosphoric acid in water (A) and methanol (B). Elution was performed as follows: 0–10 min, 10% B; 10–35 min, gradual increase of solvent B in order to achieve 100% B at 35 min; 35–45 min, 100% B. Column chromatography was performed using silica gel 60 M (0.04–0.063 mm, Merck, Darmstadt, Germany) or reversed-phase silica gel C18 (40–63 µm, Merck) as a stationary phase. TLC plates with silica gel 60 M F254 (Merck), were used to monitor fractions (DCM: MeOH (95:5 v/v) as a mobile phase). Detection was performed under UV light (254 and 366 nm) or by spraying the plates with anisaldehyde reagent. Solvents were distilled prior to use and spectral grade solvents were used for spectroscopic measurements.

#### 3.2.2. Seaweed Material

*F. spiralis* was collected in September 2012 in Peniche (Portuguese west coast) and identified as before [15]. Each sample consisted of several individuals in the same stage of development. After collection, samples were washed with NaCl 3.5%, frozen and lyophilized in a Labconco 4.5 Freezone apparatus (Kansas City, MO, USA). The dried samples were ground (particle size ≤ 910 µm) and kept in a desiccator until analysis.

#### 3.2.3. Extraction and Isolation

The freeze-dried seaweed material (dry weight: 140 g) was exhaustively extracted (1L × 3) with MeOH at room temperature. The MeOH fractions were combined and evaporated under vacuum. The dried methanolic extract was subjected to solvent-solvent partitioning to give n-hexane, EtOAc and *n*-BuOH fractions. The EtOAc fraction was evaporated to dryness (15.03 g) and 1g was taken and was subjected to C18 reversed vacuum liquid chromatography (VLC), using a step gradient starting with H_2_O:MeOH (80:20 v/v), followed by a final elution of 100% MeOH. This procedure was repeated 3 times. The less polar fraction (100% MeOH) was further submitted to consecutive silica gel 60 M column chromatography with DCM:MeOH (95:5 v/v) as a mobile phase to afford **1** (6.09 mg) and an inseparable mixture of **2** and **3** (50.14 mg) in an approximate ratio of 1:1.

#### 3.2.4. Spectrometric Data of Isolated Compounds

Spectrometric data of compounds **1**, **2** and **3** was previously reported [7,21,22].

Compound **1**: ^1^H NMR (CDCl_3_, 500 MHz): 5.35 (2H, m), 4.19 (1H, dd, *J* = 11.7, 4.5 Hz), 4.13 (1H, dd, *J* = 11.7, 6.0 Hz), 3.91 (1H, m), 3.68 (1H, dd, *J* = 11.7, 4.0 Hz), 3.58 (1H, dd, *J* = 11.7, 6.0 Hz), 2.33 (2H, t, *J* = 7.6 Hz), 2.01 (4H, m), 1.61 (2H, m), 1.29 (20H, m), 0.86 (3H, t, *J* = 6.9 Hz); ESIMS positive *m*/*z* (%) 357 [M + H]^+^ (100), 735 [2M + Na]^+^ (5), in agreement with data reported in the literature [7].

Compounds **2** and **3**: ^1^H NMR (CDCl_3_, 500 MHz) of glycerol and sugar parts, common to both compounds: 4.38 (1H, dd, *J* = 12.0, 3.3 Hz, H-1a), 4.19 (1H, dd, *J* = 12.0, 6.4 Hz, H-1b), 5.30 (1H, m, H-2), 3.89 (1H, dd, *J* = 11.0, 5.6 Hz, H-3a), 3.73 (1H, dd, *J* = 11.0, 6.4 Hz, H-3b), 4.26 (1H, d, *J* = 7.4 Hz, H-1′′′), 3.63 (1H, dd, *J* = 9.5, 7.4 Hz, H-2′′′), 3.58 (1H, dd, *J* = 9.5, 2.5 Hz, H-3′′′), 3.99 (1H, brd, *J* = 2.5 Hz, H-4′′′), 3.53 (1H, brt, *J* = 4.6 Hz, H-5′′′), 3.96 (1H, dd, *J* = 12.5, 5.8 Hz, H-6a′′′), 3.85 (1H, dd, *J* = 12.5, 4.6 Hz, H-6b′′′) and ^13^C NMR (CDCl_3_, 500 MHz): 63.1 (C-1), 70.4 (C-2), 68.5 (C-3), 104.2 (C-1′′′), 71.8 (C-2′′′), 73.6 (C-3′′′), 69.5 (C-4′′′), 74.7 (C-5′′′), 62.7 (C-6′′′), in agreement with data reported in the literature [21,22].

Compound **2**: ^1^H NMR (CDCl_3_, 500 MHz): 5.36 (18H, m, H-5′, 6′, 8′, 9′, 11′, 12′, 14′, 15′, 17′, 18′, 6′′, 7′′, 9′′, 10′′, 12′′, 13′′, 15′′, 16′′), 2.80 (14H, m, H-7′, 10′, 13′, 16′, 8′′, 11′′, 14′′), 2.31 (4H, m, H-2′, 2′′), 2.08 (2H, m, H-4′), 2.05 (6H, m, H-5′′, 17′′, 19′), 1.67 (2H, m, H-3′), 1.61 (2H, m, H-3′′), 1.37 (2H, m, H-4′′), 0.96 (6H, t, *J* = 7.5 Hz, H-18′′, 20′) and ^13^C NMR (CDCl_3_, 500 MHz): 174.1 (C-1′), 173.5 (C-1′′), 33.7 (C-2′), 34.4 (C-2′′), 25.1 (C-3′, 3′′), 26.7 (C-4′), 29.4 (C-4′′), 129.2 (C-5′), 27.1 (C-5′′), 128.1–129.0 (C-6′, 8′, 9′, 11′, 12′, 14′, 15′, 7′′, 9′′, 10′′, 12′′, 13′′), 129.8 (C-6′′), 25.8 (C-7′, 10′, 13′, 16′, 8′′, 11′′, 14′′), 127.2 (C-17′, 15′′), 132.2 (C-18′, 16′′), 20.7 (C-19′, 17′′), 14.3 (C-20′, 18′′); ESIMS positive *m*/*z* (%) 819 [M + Na]^+^ (10), 797 [M + H]^+^ (100), 635 [M + H − C_6_H_10_O_5_]^+^ (24), 617 [M + H − C_6_H_12_O_6_]^+^ (1), 333 [C_17_H_27_CO + 74]^+^ (3), 285 [C_19_H_29_CO]^+^ (0.5), in agreement with data reported in the literature [21,22].

Compound **3**: ^1^H NMR (CDCl_3_, 500 MHz): 5.36 (16H, m, H-5′, 6′, 8′, 9′, 11′, 12′, 14′, 15′, 17′, 18′, 9′′, 10′′, 12′′, 13′′, 15′′, 16′′), 2.80 (12H, m, H-7′, 10′, 13′, 16′, 11′′, 14′′), 2.31 (4H, m, H-2′, 2′′), 2.08 (2H, m, H-4′), 2.05 (4H, m, H-19′,17′′), 2.04 (2H, m, H-8′′), 1.67 (2H, m, H-3′), 1.59 (2H, m, H-3′′), 1.31 (8H, m, H-4′′, 5′′, 6′′, 7′′), 0.96 (6H, t, *J* = 7.5 Hz, H-18′′, 20′′) and ^13^C NMR (CDCl_3_, 500 MHz): 173.8 (C-1′), 173.5 (C-1′′), 33.7 (C-2′), 34.4 (C-2′′), 25.1 (C-3′), 24.9 (C-3′′), 26.7 (C-4′), 29.3 (C-4′′), 129.2 (C-5′), 29.5 (C-5′′), 128.1-129.0 (C-6′, 8′, 9′, 11′, 12′, 14′, 15′, 10′′, 12′′, 13′′), 29.8 (C-6′′), 25.8 (C-7′, 10′, 13′, 16′, 11′′, 14′′), 29.9 (C-7′′), 27.4 (C-8′′), 130.2 (C-9′′), 127.2 (C-17′, 15′′), 132.2 (C-18′, 16′′), 20.7 (C-19′, 17′′), 14.3 (C-20′, 18′′); ESIMS positive *m*/*z* (%) 821 [M + Na]^+^ (9), 799 [M + H]^+^ (100), 637 [M + H − C_6_H_10_O_5_]^+^ (23), 619 [M + H − C_6_H_12_O_6_]^+^ (1), 335 [C_17_H_29_CO + 74]^+^ (2), 285 [C_19_H_29_CO]^+^ (0.6), in agreement with data reported in the literature [21,22].

### 3.3. Anti-Inflammatory Capacity

#### 3.3.1. Cell Culture and Treatments

The murine macrophage cell line RAW 264.7 was grown at 37 °C, in DMEM supplemented with GlutaMAX™-I, 10% FBS, 100 U/L penicillin and 100 µg/mL streptomycin, in a humidified atmosphere of 5% CO_2_. Cells were inoculated at a density of 150,000 cells/well into 48-well plates and cultured until confluence. The isolated compounds were dissolved in DMSO at 100 mg/mL and stored in aliquots, at −20 °C, until analysis. Each compound was diluted with supplemented DMEM immediately before cell exposure. Cells were pre-treated with different concentrations of the isolated compounds or vehicle for 1 h. Following the addition of 1 mg/mL LPS (or vehicle) cells were further incubated for 18 h at 37 °C in a humidified atmosphere of 5% CO_2_. Dexamethasone was used as positive control. The effect of the isolated compounds was also evaluated in the absence of LPS in order to observe the changes in NO basal levels. No LPS was added to the negative controls. The final concentration of DMSO was 0.5% in all wells. Four independent assays were performed in duplicate.

#### 3.3.2. Cell Viability

##### 3.3.2.1. LDH Assay

The release of cytosolic LDH into the culture medium was used as an index of cell death. After the incubation period of 18 h at 37 °C, the culture medium was carefully removed from each well and used to determine the activity of LDH released by death cells. The oxidation of NADH during the conversion of pyruvate to lactate was used to determine LDH activity, by following the kinetic at 340 nm in a microplate reader (Multiscan ASCENT Thermo^®^, Vantaa, Finland). Results are expressed as LDH activity in the medium of exposed cells, relative to control, without extract [15].

##### 3.3.2.2. MTT Assay

Cell viability was also assessed by the mitochondria dependent reduction of MTT to formazan. After the incubation period (18 h at 37 °C), RAW 264.7 cells were washed with DPBS and then incubated for 30 min with MTT (0.5 mg/mL in DMEM). MTT is converted by mitochondrial dehydrogenases of metabolically active cells from its yellow salt to an insoluble purple formazan product, which is then solubilized with DMSO. The extent of the reduction to formazan within the cells was quantified by measuring the absorbance of the solution at 510 nm in a microplate reader (Multiscan ASCENT Thermo^®^) [15].

#### 3.3.3. NO Release by RAW 264.7 Cells

Macrophage cells release NO into the culture medium, which is converted into different nitrogen derivatives. Of them, only nitrite is stable, and for this reason, easy to measure as an indicator of NO production. After the incubation period with the test compounds (6.25–100 µg/mL for compound **1** and 15.63–500 µg/mL for the 1:1 mixture of compounds **2** and **3**) and dexamethasone (3.07–49.06 µg/mL), the nitrite accumulated in the culture medium was determined using Griess reagent [1:1 mixture (v/v) of 1% sulphanilamide and 0.1% *N*-(1-naphthyl) ethylenediamine in 2% H3PO4] [27]. Equal volumes of culture supernatant and Griess reagent were mixed and incubated for 10 min in the dark, at room temperature. The absorbance of the chromophore formed during the diazotization of nitrite with sulphanilamide and subsequent coupling with naphthylethylenediamine dichloride was read at 562 nm in a microplate reader (Multiscan ASCENT Thermo^®^). Control values were obtained in the absence of the isolated compounds and after addition of LPS.

### 3.4. Statistical Analysis

Data were analysed by using GraphPad PRISM software (GraphPad software, San Diego, CA, USA) (version 5.2 for Windows). One-way analysis of variance (ANOVA), using the Dunnet Multiple Comparison test was carried out on data obtained from four independent assays performed in duplicate for each sample. Levels of statistical significance at *p* < 0.05, *p* < 0.01 and *p* < 0.001 were used.

## 4. Conclusions

In the present work, the glyceroglycolipids composition of *F. spiralis* was investigated. Two MGDGs and one monoacylglycerol were isolated and characterized for the first time in this species. The anti-inflammatory capacity of the isolated compounds was also described herein for the first time. The glyceroglycolipids isolated from *F. spiralis* are mainly composed of polyunsaturated fatty acids. These compounds display anti-inflammatory activity by inhibiting NO release by macrophages.

A combination of our study with previous investigations involving similar naturally occurring glyceroglycolipids [8,9,11] highlights their anti-inflammatory capacity over different types of cell lines. This contributes to promote their use as potential naturally occurring templates for future drug development.
